# Plasmatic exosome-derived circRNAs panel act as fingerprint for glioblastoma

**DOI:** 10.18632/aging.203368

**Published:** 2021-08-12

**Authors:** Dongyan Xia, Xuhui Gu

**Affiliations:** 1 Department of Neurosurgery, Haimen People's Hospital, Nantong 226100, Jiangsu Province, China

**Keywords:** circRNA, plasma, risk score analysis, exosome, glioblastoma multiforme

## Abstract

Circular RNAs (circRNAs) have recently emerged as a new class of RNAs, highly enriched in the brain and very stable within cells, exosomes and body fluids. In this study, we aimed to screen the exosome derived circRNAs in glioblastoma multiforme (GBM) and investigate whether these circRNAs could predict GBM as potential biomarkers. The exosome was extracted from the plasma of GBM patients and healthy volunteers and validated by immunoblotting. The circRNA microarray was employed with three samples in each group to screen the dysregulated circRNAs isolated from the exosome. Five circRNAs were first selected as candidates with the upregulated level in exosome isolated from the plasma of GBM. Further validation found that only hsa_circ_0055202, hsa_circ_0074920 and hsa_circ_0043722 were consistent with training set. The Receiver operating characteristic (ROC) curve also revealed a high diagnostic ability an area under ROC curve value (AUC) for single circRNA and combined. The AUC for hsa_circ_0055202, hsa_circ_0074920, hsa_circ_0043722 and the combined was 0.810, 0.670, 0.938 and 0.988 in training set. For the validation set, the AUC was 0.850, 0.625, 0.750 and 0.925. The three circRNAs were further investigated with stable expression in human plasma samples. In conclusion, the exosome derived hsa_circ_0055202, hsa_circ_0074920 and hsa_circ_0043722 might be the potential biomarker for predicting the GBM.

## INTRODUCTION

As a common tumor of the nervous system, glioma accounts for 80% of the primary tumors of the brain. The malignant degree of glioma is high, and the overall prognosis and survival are poor [1, 2]. With the deepening of the research on glioma, there have been a variety of treatment methods including surgical treatment, chemotherapy, radiotherapy and so on, which have improved the prognosis survival time of glioma to a certain extent, but the prognosis of glioblastoma is still relatively poor. Statistically, the 3-year survival rate for GBM is less than 5% [3, 4]. Glioblastoma is a kind of glioma with high degree of malignancy, and it is also the primary disease of death related diseases caused by nerve cell malignancy [5, 6]. Increasing evidence has proved that although multiple approaches have been developed such as surgical resection, radiotherapy and postoperative chemotherapy, the disease prognosis remains poor [7]. Researchers have identified that prognosis would be better if GBM was diagnosed at an earlier stage [8]. Thus, the early diagnosis or novel therapeutic target for GBM is very necessary.

With the development of second-generation sequencing technology, more and more non-coding RNAs have been discovered, and it has been proved that these non-coding RNAs are involved in a variety of biological functions in human diseases and play an important role. Circular RNAs (circRNAs) are one of the new members of non-coding RNAs that have been discovered recently. Their structure has certain particularity and they form single-stranded closed circRNAs [9, 10]. Furthermore, researchers have indicated the differential circRNA profiles of the serum exosomes derived from patients with other human malignant tumors, such as gastric cancer, colon cancer and hepatocellular carcinoma [11, 12]. CircRNAs have a circular structure and are relatively stable. Studies have shown that the special structure of circRNAs can avoid their degradation [13].

Exosomes are recently identified as one of the important components of cellular fluid, which are mainly secreted extracellular through cellular vesicles. Studies have shown that exosomes play an important mediating role in cell-to-cell cross-talk and substance exchange. In addition, a growing number of studies have shown that exosomes can also be used as a new molecular diagnostic marker, which plays an important role in the early diagnosis of diseases [14]. In addition, exosomes express major tissue phase endogenous molecules, such as capacitive complex (MHC) and CD63, and contain a large number of specific proteins and functional mRNAs, miRNAs, and even DNA. In addition, researchers also extracted abundant circRNAs from exosomes, and confirmed that the expression of circRNAs in exosomes and intracellular circRNAs was consistent [15]. In addition, previous studies also showed that there were significantly differentially expressed circRNA lineages in peripheral blood exosomes of some tumor patients, suggesting that it is possible to detect specific circRNA after peripheral blood exosomes were extracted, which may be used as molecular markers for the subsequent diagnosis or treatment of this disease [16, 17]. However, little evidence was investigated about the circRNA in the exosome of GBM patients.

In this study, the circRNA microarray was employed to screen the dysregulated circRNAs in the exosome extracted from plasma samples of GBM. A multiple staged validation including a training group and validation group was followed to test whether the abnormal expressed plasma-derived circRNAs might predict the GBM from healthy volunteers.

## RESULTS

### Subject characteristics

After analyzing the clinical information, we found no difference in the age and gender distribution for the GBM comparing with the healthy controls. Of the 120 GBM patients, 71 patients were proved with tumor size ≤3cm(diameter), the rest 49 was above 3cm. The tumor was infratentorial in 31 patients, while 89 patients' tumors were supratentorial. According to the WHO grade of GBM, 45 patients were labelled with I-II grade and 75 patients were labelled with III-IV grade (Table 1).

**Table 1:** Clinicopathological features analysis of glioblastoma (GBM) patients and cancer-free control samples.

	**Control**	**GBM**	***p* value**
Total number	120	120	
Age Mean (SE) year	52.01(0.33)	51.79(0.22)	0.35^a^
Sex (male/female)	72/48	76/44	0.59^b^
Tumor Size(cm)			
≤3cm		71	
>3cm		49	
Tumor location			
Infratentorial		31	
Supratentorial		89	
WHO grade			
I–II		45	
III–IV		75	

^a^Student t-test.

^b^Chi-square test.

### Exosome identification and circRNA expression profile in GBM and control group

Exosome specific markers such as CD9, CD63 and TSG101 were used as positive markers and calnexin was used as negative markers to detect human plasma samples.

**Figure 1:**
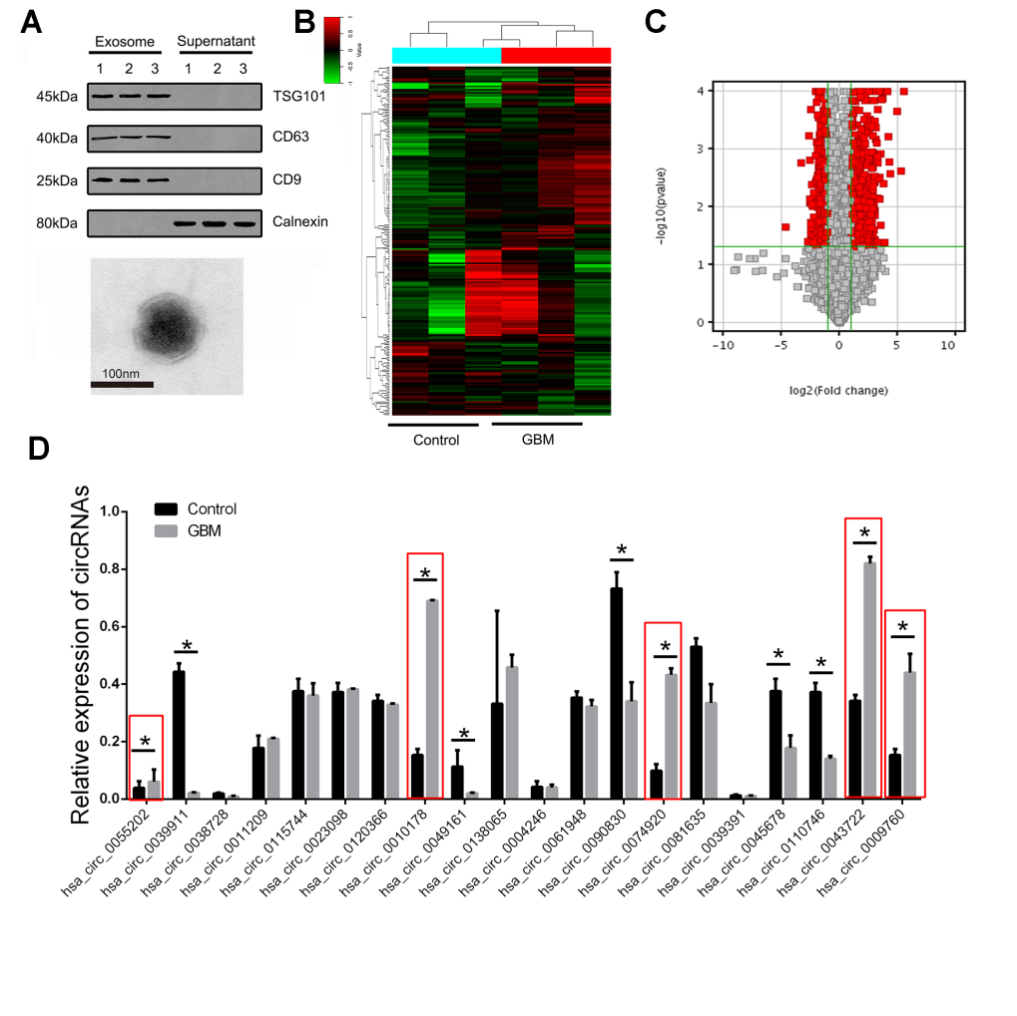
**The expression landscape of circulating exosome-derived circRNAs in GBM patients.** (**A**) Western blot was used to detect the expression of CD9, CD63, TSG101 and calnexin in exosome isolated from plasma samples of GBM patients (upper panel). Plasma exosomes were observed using TEM (lower panel). (**B**) Cluster analysis of the different expression of the circRNAs in different groups. (**C**) Volcano plot presented the dysregulated circRNAs in GBM comparing with control group. Red plots indicated with significant difference. (**D**) The expression of circRNAs were confirmed by RT-PCR in training set. * indicated P<0.05, n.s. indicated no significance.

We first confirmed the abundant levels of exosomes in plasma samples. In addition, we also confirmed the morphology of exosomes as irregular planets with a diameter span of 30-100nm under projection electron microscopy and with a clear outer membrane structure. (Figure 1A). After extracting the total RNA from the exosome, the circRNA microarray was further employed for high-throughput detection. Three samples from each group were randomly selected for microarray detection. As shown in Figure 1B, we screened the differentially expressed circRNAs with 2/0.25 as the fold change cut-off, and found that there were significantly different circRNA expression profiles in the plasma exosomes of patients in the GBM group compared with the healthy controls. Furthermore, P<0.05, CT value <35 and >75% of the sample detection efficiency were selected as the screening parameters. Finally, 102 circRNAs with abnormally high expression and 67 circRNAs with abnormally low expression were obtained as the targets for subsequent studies (Figure 1C).

Abnormal elevation of the index is usually selected as the traditional diagnostic marker. In this study, in order to further screen potential biological markers for GBM, we selected the top 20 differential multiples in the above abnormally high expression as the targets for further verification.

### Multiple staged validation of differentially expressed circRNAs in the training group and validation group

Multistage validation was performed for the 20 circRNAs included above. 20 randomly selected pairs of GBM patients and 20 pairs of healthy control plasma exosomes were selected as the test group for the first batch of screening, followed by further validation of the remaining 100 pairs of samples as the validation group. We found statistically significant differences in 10 of the 20 circRNAs in the test group. Further analysis showed that 5 circRNAs were expressed in a state consistent with the microarray results, while the remaining 5 circRNAs were presented with lower expression in GBM group (Figure 1D).

**Figure 2:**
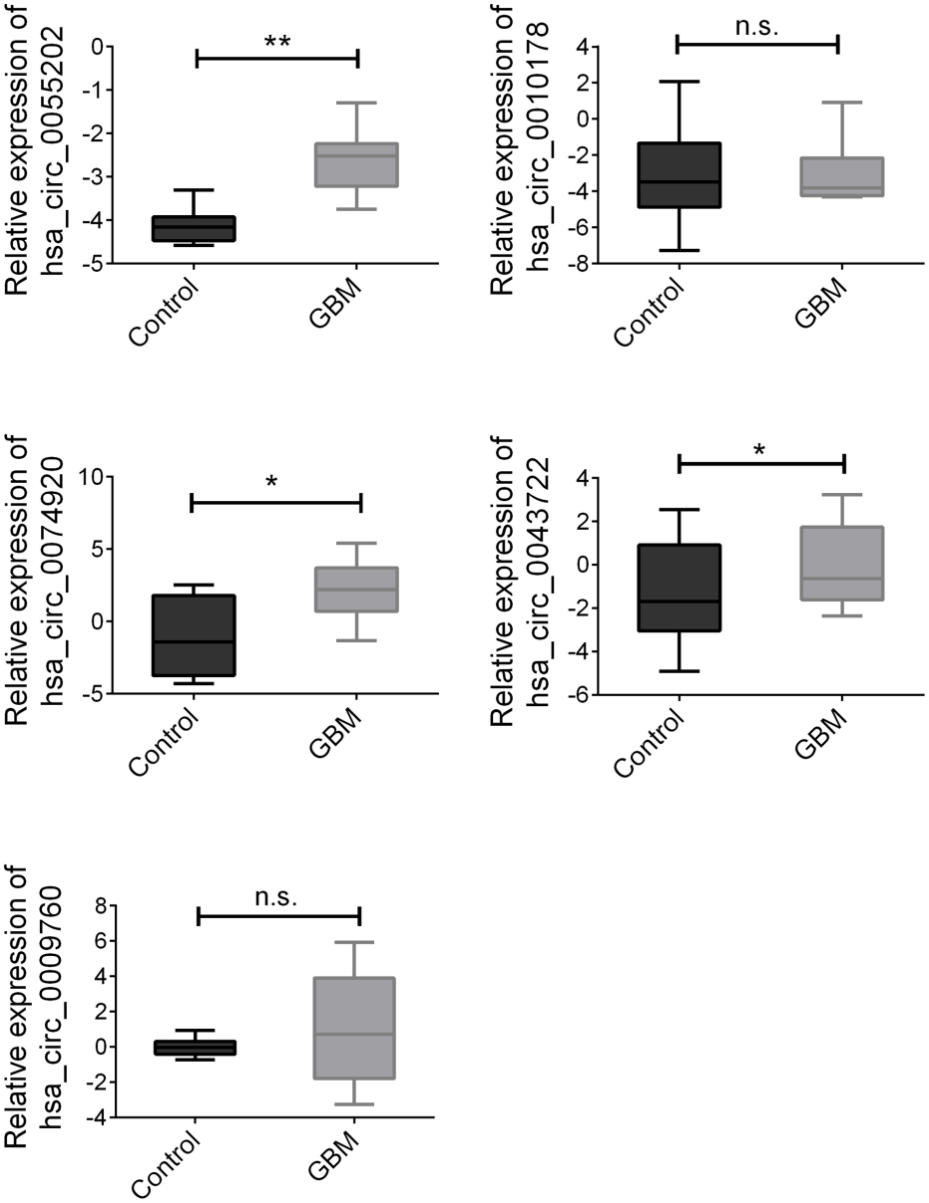
**Validation of three candidate circRNAs.** Plasma from 100 paired GBM and healthy controls were enrolled. Data were presented as plot of the median and range of log-transformed relative expression level. * indicated p<0.05; ** indicated p < 0.01, n.s. indicated no significance.

According to the data of the above training group, the rest 100 pairs of GBM and healthy control group samples were used for further verification. We detected the above circRNAs with upregulated expression and found that hsa_circ_0055202, hsa_circ_0074920 and hsa_circ_0043722 were still expressed in an elevated state in GBM. The expression of hsa_circ_0009760 was high in GBM, but there was no statistical difference. There was no significant difference in the expression of hsa_circ_0010178 (Figure 2).

### Risk score analysis and diagnosis ability investigation

**Figure 3:**
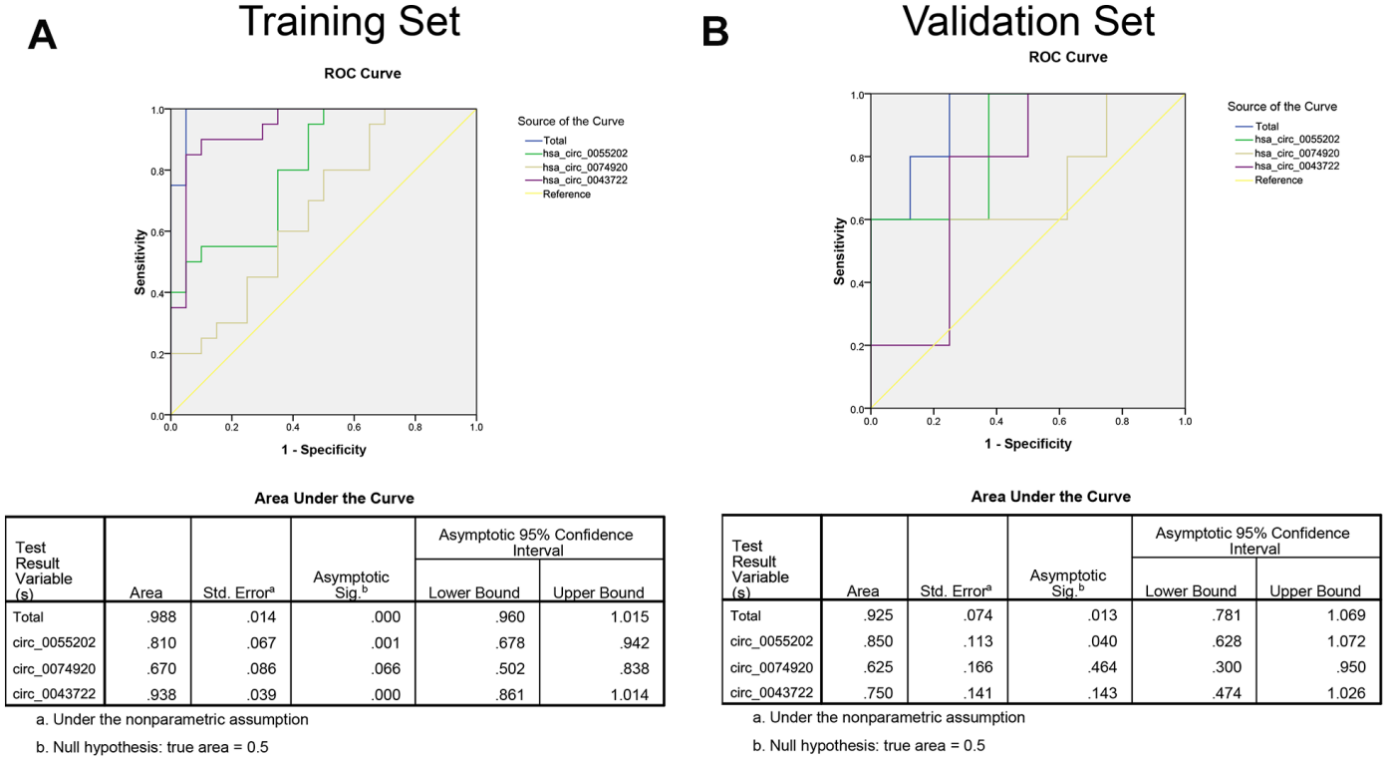
**The ROC curve based on risk score analysis for predicting GBM from healthy population.** (**A**) ROC curve analysis was conducted for discrimination between GBM patients and control group in training set. Each group including 20 samples. (**B**) ROC curve analysis was conducted for discrimination between GBM patients and control group in validation set. Each group including 100 samples.

To further analyze the potential predicting ability of GBM from healthy controls. Receiver operating characteristic curve (ROC) was used to calculate the specific sensitivity and specificity. Firstly, the isolated circRNAs and three combined factors were analyzed in the training set (20 samples in each group as described above). As presented in Figure 3A, the areas under the curve (AUC) of hsa_circ_0055202, hsa_circ_0074920, hsa_circ_0043722 and combination of circRNAs (Merged) were 0.810, 0.670. 0.938 and 0.988, respectively. We continued the analysis using the same method in the validation group. As presented in Figure 3B, in validation group, the AUC of the four circRNAs and merged were 0.850, 0.625. 0.750 and 0.925, respectively.

We defined the cut-off value as the maximum of sensitivity and specificity and calculated the positive predictive value (PPV) and negative predictive value (NPV). To distinguish between GBM and the control group, PPV and NPV in the training set were 90% and 90%, respectively. By using the same method in the validation set, we obtained the PPV and NPV were 84% and 86%, respectively (Table 2).

**Table 2:** Risk score analysis of in different groups.

**Score**	**0-4.421**	**4.421-7.339**	**NPV^a^**	**PPV^b^**
Training set(n=20)			0.90	0.90
GBM	2	18		
Control	18	2		
Validation set(n=100)			0.84	0.86
GBM	17	83		
Control	87	13		

^a^PPV, positive predictive value.

^b^NPV, negative predictive value.

### Stability detection of circRNAs in plasma samples

**Figure 4:**
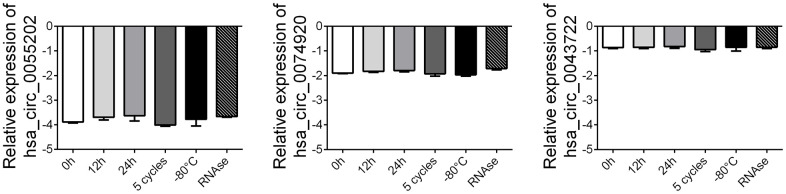
**Stability detection of the four circRNAs in human plasma.** RT-qPCR was applied for detecting the expression level of the three circRNAs. Data was presented as mean ± SEM with log-transformed. No significant difference was observed in each group.

To further verify the stability of circRNA, we randomly selected 5 healthy control plasma samples and subjected the above samples to different treatments, including storage at -80° C for 7 days, storage at room temperature for 12 hours and 24 hours, repeated freeze-thaw treatment for 5 times and digestion with RNase enzyme. After different treatments, the exosome RNA was extracted and amplified by RT-PCR, and the changes of circRNA expression among the groups were compared. The results showed that the expression of circ_0055202, circ_0074920 and circ_0043722 did not significantly change after the above different treatments, suggesting that the expression of circ_0055202, circ_0074920 and circ_0043722 were relatively stable and detectable in plasma (Figure 4).

### The hsa_circ_0055202, hsa_circ_0074920 and hsa_circ_0043722 could promote the proliferation of GBM cells *in vitro*

**Figure 5:**
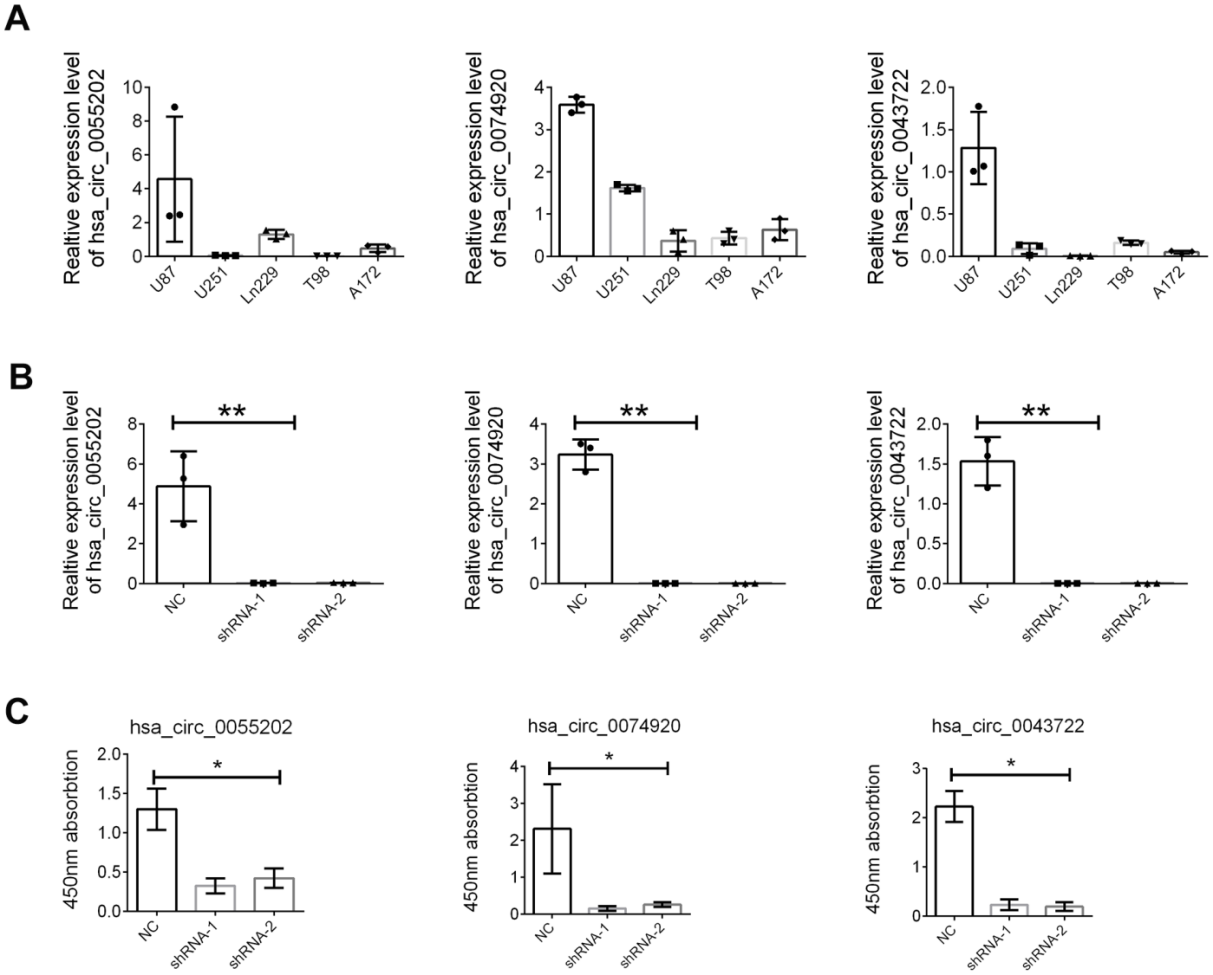
**The hsa_circ_0055202, hsa_circ_0074920 and hsa_circ_0043722 promoted cell proliferation of GBM cell lines.** (**A**) Relative expression of hsa_circ_0055202, hsa_circ_0074920 and hsa_circ_0043722 in GBM cell lines. (**B**) Relative expression of hsa_circ_0055202, hsa_circ_0074920 and hsa_circ_0043722 in cells treated with shRNA. (**C**) CCK8 assay in cells treated with circRNA shRNA. * indicated P < 0.05, ** indicated P < 0.01.

The detailed function of the three circRNA including circ_0055202, circ_0074920 and circ_0043722 were further investigated. Firstly, as presented in Figure 5A, the expression abundance of the there circRNA were examined in GBM cells including U87, U251, Ln229, T98, and A172. Based on this, we chose the U87 cells for further function assay with a relatively high abundance level. We knocked down the three circRNAs through the shRNA technology and confirmed the suppression of the three circRNA in U87 (Figure 5B). The CCK8 assay and transwell assay was employed. We found that either suppression of circ_0055202, circ_0074920 or circ_0043722 could reduce the proliferation of GBM cells (Figure 5C); however, no significance was found in cell invasion (Supplementary Figure 1).

## DISCUSSION

Glioblastoma (GBM) is a malignant brain cancer characterized by aggressive growth, rich vasculature, distant metastasis and poor prognosis [18]. Advanced treatments have improved therapeutic outcomes. However, GBM vascularization and blood-brain barrier (BBB) could block the penetration of anti-tumor agents, reducing the treatment efficiency [19]. Therefore, there will be remarkable significance to explore early diagnostic markers related to the occurrence of GBM for improving the efficiency of early diagnosis and prognosis of GBM.

Previous studies have shown that circRNAs were generally formed after splicing in the nucleus, as well as in the linear state of splicing and transcription. The subcellular localization of circRNA was generally located in the cytoplasm [20]. Researchers have found that circRNAs played various roles in the development and progression of glioblastoma and was involved in various biological processes such as cell growth and metastasis. It has been reported that a large number of circRNAs act as sponges to mediate miRNAs and their target genes, adsorbing miRNAs, and then regulate the expression level of target genes. Some circRNAs could directly and specifically bind to RNA-binding proteins to regulate the functions of RNA-binding proteins. Consistent with the function of traditional circRNAs, circentPD7 expressed in glioblastoma cells, and was participated in the process of tumor metastasis by mediating cell invasion ability. In GBM, circentPD7 has been proved to be able to specifically adsorb miR-101-3p, thus affecting the expression of its downstream target genes [21].

For the biomarker exploration, some circRNAs have also proved might be a potential biomarker for GBM. Chen, et al conducted the experiment by using published circRNA in tumor tissues to screen the potential biomarker for GBM. It was found that three circRNAs (circFOXO3, circ_0029426 and circ-SHPRH), which have been identified to have certain functions in GBM, were significantly differentially expressed in GBM, and may serve as potential biological markers of GBM [22]. However, this study was restricted by published article and without high throughput investigation.

For circRNA, recent study has shown that circRNA can be specifically presented in exosomes and detected [23]. It was found that circRNAs expressed in exosomes differed from those in other suborganelles. CircRNAs specifically expressed in exosomes are usually medium in length and relatively low in GC content. In addition, circRNAs in exosomes can often participate in cell-to- cell “communication” by acting as intermediators [24, 25].

The exosomal circRNAs have been used as potential biomarkers to screen gastrointestinal cancers including pancreatic cancer, colorectal cancer and gastric cancer [26]. Similarly, detection of exosomal circRNA_0056616 expression in plasma showed that circRNA_0056616 could be used as a current marker of lung metastasis, suggesting early lymph node metastasis of lung adenocarcinoma [27]. However, little was known regarding the detailed expression level of circRNA or mechanism investigation was found in the plasma exosome of GBM patients.

In conclusion, we identified three unique circRNAs as potential biomarkers for early screening for GBM in healthy populations and might provide a new minimally invasive method for diagnosis and dynamic monitoring of GBM.

## MATERIALS AND METHODS

### Subjects

We conducted the retrospective case-control study by enrolling total 120 GBM patients and paired 120 healthy controls. All the patients were diagnosed histologically in Haimen People’s Hospital range from June 2015 to May 2019. This study was approved by an institutional review board of the affiliated Haimen People’s Hospital (HMPH_IRB_01152). Written informed consents were obtained from all enrolled subjects. All plasma samples were collected using EDTA anticoagulant tubes. Half an hour after sampling, the samples were centrifuged at 3000 rpm for 10 minutes. The upper plasma was absorbed and stored at -80° C. All patients signed informed consent before sample collection. The relevant clinical data of all patients was summarized in Table 1. All experiments were performed in compliance with government policies and the Helsinki Declaration. All subjects were informed about the study and provided their consent prior to specimen collection.

### Exosome isolation and identification

Exoquick Exosome precipitate solution (System Biosciences, Mountain View, CA, USA) was used to extract exosomes from plasma samples. Exosomes were efficiently isolated using SBI’s ExoQuick/ExoQuick-TC solutions, resuspend exosome pellet in 350 μl of Lysis Buffer. Further, biomarker detection and morphological identification were carried out for the extracted exosomes. Exosome biomarkers including CD9, CD63 and TSG101 were first used to detect their expression levels. In addition, calnexin was used as negative control. Then the morphology of exosomes extracted was observed by transmission electron microscopy (TEM).

### RNA extraction and quantitative real-time PCR (qRT-PCR)

We used Trizol (Invitrogen, CA, USA) method for RNA extraction, and the specific method was carried out according to its instructions. After RNA extraction, its concentration and purity were determined by ND-1000 (Thermo, CA, USA), and RNA was clarified by 1% agarose electrophoresis to ensure RNA integrity. In order to clarify the stability of circRNA expression, we used the combination of internal and external parameters for correction. The internal reference was GAPDH, and the external reference was cel-miR-39 (ABI, CA, USA). The circRNA was amplified by random primer method and amplified in ABI 7900 (ABI, CA, USA). The relative expression of circRNA was calculated using the 2^-ΔΔCq^ method.

### circRNA microarray

The microarray-based circRNA expression profiling was conducted by Capitalbio (Beijing, China) using plasma RNA samples. We conducted the sample preparation and microarray hybridization in accordance with instrument maufacture's manual (Arraystar, Inc). Briefly, samples were enriched for circRNAs by treating them with RNase R to degrade linear RNA, after which random primers were used to reverse transcribe enriched circRNAs into cDNA. This reduces the initial 37,681 input genes in each sample to about 15,000 detected genes. A bilateral Mann-Whitney U test performed by Wilcox was employed. Finally, we use the Benjamin-Hochberg correction (R. P. Adojust function) to explain the multiple tests.

### Multiple phase validation

The screening phase was divided into training group and validation group. 20 samples each group were enrolled in training group while the validation group contained another 100 samples in each group. Groups were named as healthy volunteers and GBM patients.

Groups were named as healthy volunteers and GBM patients. Risk scores analysis was used to analyze the predictive power of certain circRNAs. In brief, the upper 95% reference interval (95%CI) of each circRNA value in the control group was used as the cut-off value for a certain circRNA expression level. If circRNA expression was higher than 95% CI in this sample, we rated it as 1, and if it was lower than 95% CI, we rated it as 0. The risk score was defined as a linear combination of the expression levels of each circRNA. The risk score for the circRNAs was calculated using weight regression coefficients derived from univariate logistic regression analyses for each circRNAs. The samples were ranked by RSF and then divided into a high-risk group (representing the GBM group) and a low-risk group (representing the predictive control individuals).

### Cell lines and cell culture

GBM cell lines, including U87, U251, Ln229, T98, and A172 cells, were purchased from the Cell Bank of Type Culture Collection of the Chinese Academy of Sciences (Shanghai, China), and were cultured and stored according to the guidelines of the cell bank. The culture medium used was Dulbecco’s modified Eagle’s medium (DMEM; Winsent, Quebec, Canada) containing 10% fetal bovine serum (FBS), 100 U/ml penicillin, and 100 μg/ml streptomycin. All the cell lines were incubated in a 5% CO2 humidified incubator at 37° C.

### Cell proliferation assay

In each well of a 96-well plate, 1000 cells were plated in total. The CCK-8 reagent (Dojindo Seed, Japan) was added directly to the culture medium at the stated time (24 h, 48 h, 72 h, 96 h). Cells were then incubated at 37° C for 2 hours, and the optional density (OD) value was measured at 450 nm by microplate reader (BioTek Instruments, USA). These studies were carried out three times.

### Transwell assay

Cells were seeded in upper chambers with 200ul of serum-free medium for Transwell assays. The transwell chamber (Corning, USA) was paved with matrigel mix (BD Biosciences, USA) for invasion assays. The bottom chamber medium contained 10% FBS to attract upper cells. The upper chambers were set and stained for 20 minutes with a 0.5% crystal violet solution after a 48-hour incubation period. The cell lines were photographed and counted in five distinct regions.

### Statistical analysis

SPSS 20.0 (IBM, SPSS, Chicago, USA) and GraphPad Prism 5 were used for statistical analysis. To explore whether two or more classes were statistically significant, we used the Student's t-test and one-way ANOVA. The data was interpreted as Mean±SEM. For all studies, P<0.05 between groups were considered statistically significant.

### Availability of data and materials

The datasets used and/or analyzed during the current study are available from the corresponding author on reasonable request.

## Supplementary Material

Supplementary Figure 1

## References

[1] Ji J, Zhao L, Zhao X, Li Q, An Y, Li L, Li D. Genome-wide DNA methylation regulation analysis of long non-coding RNAs in glioblastoma. Int J Mol Med. 2020; 46:224–38. 10.3892/ijmm.2020.4579 32319552 PMC7255472

[2] Szulzewsky F, Arora S, de Witte L, Ulas T, Markovic D, Schultze JL, Holland EC, Synowitz M, Wolf SA, Kettenmann H. Human glioblastoma-associated microglia/monocytes express a distinct RNA profile compared to human control and murine samples. Glia. 2016; 64:1416–36. 10.1002/glia.23014 27312099

[3] Bush NA, Chang SM, Berger MS. Current and future strategies for treatment of glioma. Neurosurg Rev. 2017; 40:1–14. 10.1007/s10143-016-0709-8 27085859

[4] Pace A, Dirven L, Koekkoek JA, Golla H, Fleming J, Rudà R, Marosi C, Le Rhun E, Grant R, Oliver K, Oberg I, Bulbeck HJ, Rooney AG, et al, and European Association of Neuro-Oncology palliative care task force. European Association for Neuro-Oncology (EANO) guidelines for palliative care in adults with glioma. Lancet Oncol. 2017; 18:e330–40. 10.1016/S1470-2045(17)30345-5 28593859

[5] Mellinghoff IK, Ellingson BM, Touat M, Maher E, De La Fuente MI, Holdhoff M, Cote GM, Burris H, Janku F, Young RJ, Huang R, Jiang L, Choe S, et al. Ivosidenib in Isocitrate Dehydrogenase 1 - Mutated Advanced Glioma. J Clin Oncol. 2020; 38:3398–406. 10.1200/JCO.19.03327 32530764 PMC7527160

[6] Juškys R, Chomanskis Ž. Glioblastoma Following Traumatic Brain Injury: Case Report and Literature Review. Cureus. 2020; 12:e8019. 10.7759/cureus.8019 32528758 PMC7282376

[7] Khasraw M, Reardon DA, Weller M, Sampson JH. PD-1 Inhibitors: Do they have a Future in the Treatment of Glioblastoma? Clin Cancer Res. 2020; 26:5287–96. 10.1158/1078-0432.CCR-20-1135 32527943 PMC7682636

[8] Lei K, Xia Y, Wang XC, Ahn EH, Jin L, Ye K. C/EBPβ mediates NQO1 and GSTP1 anti-oxidative reductases expression in glioblastoma, promoting brain tumor proliferation. Redox Biol. 2020; 34:101578. 10.1016/j.redox.2020.101578 32526700 PMC7287278

[9] Migita K, Komori A, Kozuru H, Jiuchi Y, Nakamura M, Yasunami M, Furukawa H, Abiru S, Yamasaki K, Nagaoka S, Hashimoto S, Bekki S, Kamitsukasa H, et al. Circulating microRNA Profiles in Patients with Type-1 Autoimmune Hepatitis. PLoS One. 2015; 10:e0136908. 10.1371/journal.pone.0136908 26575387 PMC4648542

[10] Tong YS, Wang XW, Zhou XL, Liu ZH, Yang TX, Shi WH, Xie HW, Lv J, Wu QQ, Cao XF. Identification of the long non-coding RNA POU3F3 in plasma as a novel biomarker for diagnosis of esophageal squamous cell carcinoma. Mol Cancer. 2015; 14:3. 10.1186/1476-4598-14-3 25608466 PMC4631113

[11] Zhang S, Song G, Yuan J, Qiao S, Xu S, Si Z, Yang Y, Xu X, Wang A. Circular RNA circ_0003204 inhibits proliferation, migration and tube formation of endothelial cell in atherosclerosis via miR-370-3p/TGFβR2/phosph-SMAD3 axis. J Biomed Sci. 2020; 27:11. 10.1186/s12929-019-0595-9 31900142 PMC6941276

[12] Goodall GJ, Wickramasinghe VO. RNA in cancer. Nat Rev Cancer. 2021; 21:22–36. 10.1038/s41568-020-00306-0 33082563

[13] Li X, Diao H. Circular RNA circ_0001946 acts as a competing endogenous RNA to inhibit glioblastoma progression by modulating miR-671-5p and CDR1. J Cell Physiol. 2019; 234:13807–19. 10.1002/jcp.28061 30663767 PMC13483025

[14] Qi H, Liu C, Long L, Ren Y, Zhang S, Chang X, Qian X, Jia H, Zhao J, Sun J, Hou X, Yuan X, Kang C. Blood Exosomes Endowed with Magnetic and Targeting Properties for Cancer Therapy. ACS Nano. 2016; 10:3323–33. 10.1021/acsnano.5b06939 26938862

[15] LeBleu VS, Kalluri R. Exosomes Exercise Inhibition of Anti-Tumor Immunity during Chemotherapy. Immunity. 2019; 50:547–49. 10.1016/j.immuni.2019.02.019 30893584

[16] Wang HX, Huang QL, Shen JY, Xu T, Hong F, Gong ZY, Li F, Yan Y, Chen JX. Expression profile of circular RNAs in IDH-wild type glioblastoma tissues. Clin Neurol Neurosurg. 2018; 171:168–73. 10.1016/j.clineuro.2018.06.020 29920451

[17] Xu H, Zhang Y, Qi L, Ding L, Jiang H, Yu H. NFIX Circular RNA Promotes Glioma Progression by Regulating miR-34a-5p via Notch Signaling Pathway. Front Mol Neurosci. 2018; 11:225. 10.3389/fnmol.2018.00225 30072869 PMC6058096

[18] Goranci-Buzhala G, Mariappan A, Gabriel E, Ramani A, Ricci-Vitiani L, Buccarelli M, D’Alessandris QG, Pallini R, Gopalakrishnan J. Rapid and Efficient Invasion Assay of Glioblastoma in Human Brain Organoids. Cell Rep. 2020; 31:107738. 10.1016/j.celrep.2020.107738 32521263

[19] Baron RB, Lakomkin N, Schupper AJ, Nistal D, Nael K, Price G, Hadjipanayis CG. Postoperative outcomes following glioblastoma resection using a robot-assisted digital surgical exoscope: a case series. J Neurooncol. 2020; 148:519–27. 10.1007/s11060-020-03543-3 32519286

[20] Barbagallo D, Caponnetto A, Cirnigliaro M, Brex D, Barbagallo C, D’Angeli F, Morrone A, Caltabiano R, Barbagallo GM, Ragusa M, Di Pietro C, Hansen TB, Purrello M. CircSMARCA5 Inhibits Migration of Glioblastoma Multiforme Cells by Regulating a Molecular Axis Involving Splicing Factors SRSF1/SRSF3/PTB. Int J Mol Sci. 2018; 19:480. 10.3390/ijms19020480 29415469 PMC5855702

[21] Zhu F, Cheng C, Qin H, Wang H, Yu H. A novel circular RNA circENTPD7 contributes to glioblastoma progression by targeting ROS1. Cancer Cell Int. 2020; 20:118. 10.1186/s12935-020-01208-9 32308563 PMC7147020

[22] Chen A, Zhong L, Ju K, Lu T, Lv J, Cao H. Plasmatic circRNA Predicting the Occurrence of Human Glioblastoma. Cancer Manag Res. 2020; 12:2917–23. 10.2147/CMAR.S248621 32425605 PMC7196774

[23] Hu W, Liu C, Bi ZY, Zhou Q, Zhang H, Li LL, Zhang J, Zhu W, Song YY, Zhang F, Yang HM, Bi YY, He QQ, et al. Comprehensive landscape of extracellular vesicle-derived RNAs in cancer initiation, progression, metastasis and cancer immunology. Mol Cancer. 2020; 19:102. 10.1186/s12943-020-01199-1 32503543 PMC7273667

[24] Sun J, Li B, Shu C, Ma Q, Wang J. Functions and clinical significance of circular RNAs in glioma. Mol Cancer. 2020; 19:34. 10.1186/s12943-019-1121-0 32061256 PMC7023692

[25] Chen W, Quan Y, Fan S, Wang H, Liang J, Huang L, Chen L, Liu Q, He P, Ye Y. Exosome-transmitted circular RNA hsa_circ_0051443 suppresses hepatocellular carcinoma progression. Cancer Lett. 2020; 475:119–28. 10.1016/j.canlet.2020.01.022 32014458

[26] Wang Y, Li Z, Xu S, Guo J. Novel potential tumor biomarkers: Circular RNAs and exosomal circular RNAs in gastrointestinal malignancies. J Clin Lab Anal. 2020; 34:e23359. 10.1002/jcla.23359 32419229 PMC7370736

[27] He F, Zhong X, Lin Z, Lin J, Qiu M, Li X, Hu Z. Plasma exo-hsa_circRNA_0056616: A potential biomarker for lymph node metastasis in lung adenocarcinoma. J Cancer. 2020; 11:4037–46. 10.7150/jca.30360 32368286 PMC7196257

