# Supplementary material for: Plasmatic exosome-derived circRNAs panel act as fingerprint for glioblastoma
Source: Aging (Albany NY). 2021 Aug 12;13(15):19575–86. doi: 10.18632/aging.203368 (PMC8386567; doi:10.18632/aging.203368)
Supplement: Supplementary Figure 1 [file aging-13-203368-s001.pdf]

SUPPLEMENTARY MATERIALS

Supplementary Figure

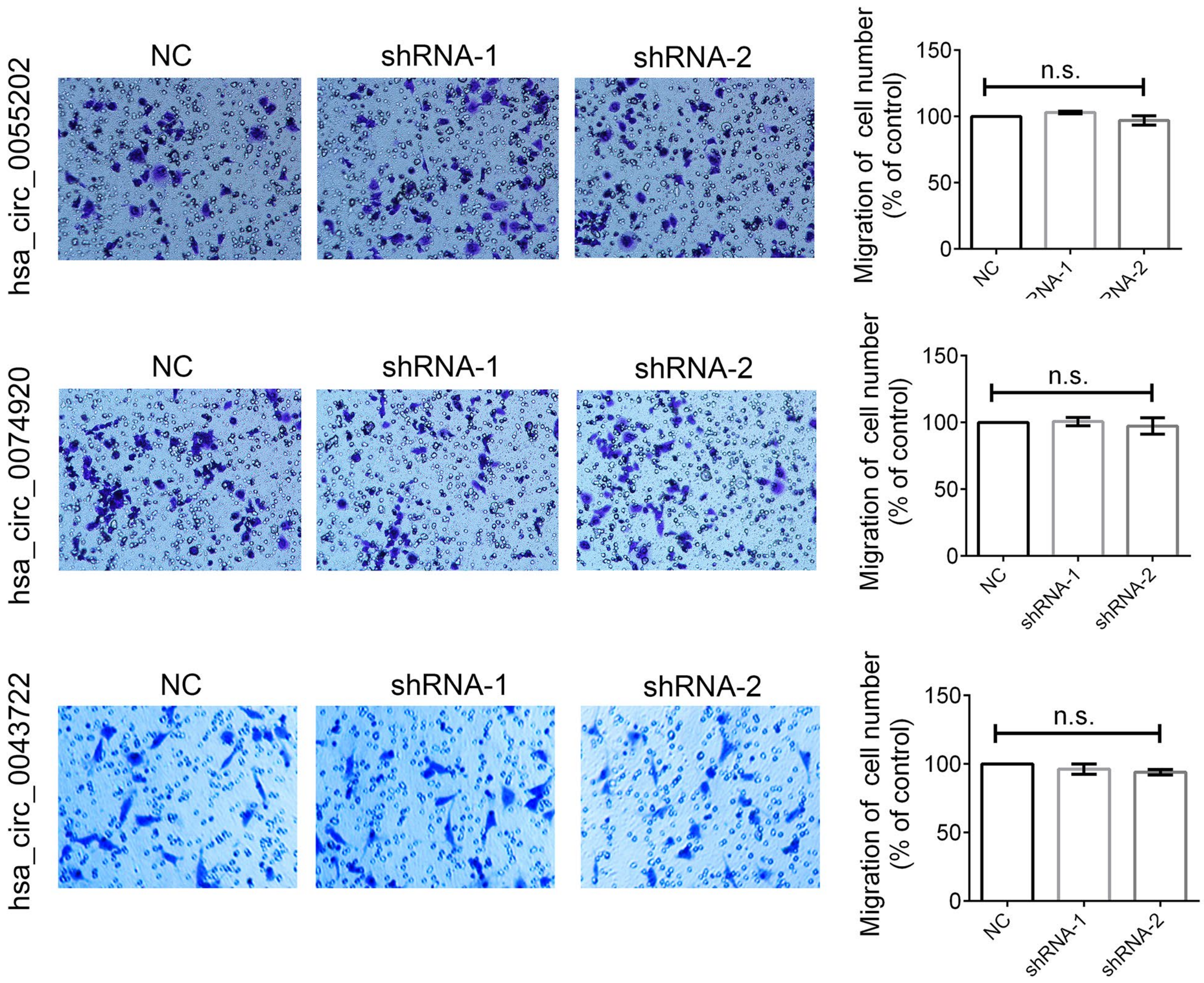

Supplementary Figure 1. The hsa_circ_0055202, hsa_circ_0074920 and hsa_circ_0043722 promoted cell proliferation of GBM cell lines was not involved in cell invasion. n.s. indicated no significant.
